# The Double-Edged Sword of Iranian Social Media Against COVID-19

**DOI:** 10.1017/S1049023X20000722

**Published:** 2020-05-21

**Authors:** Mahmoudreza Peyravi, Milad Ahmadi Marzaleh

**Affiliations:** 1. Assistant Professor, Department of Health in Disasters and Emergencies, Health Human Resources Research Center, School of Management and Medical Informatics, Shiraz University of Medical Sciences, Shiraz, Iran; 2. Assistant Professor, Research Center for Emergency and Disaster Resilience, Red Crescent Society of the Islamic Republic of Iran, Tehran, Iran; 3. PhD of Health in Disasters and Emergencies, Student Research Committee, Department of Health in Disasters and Emergencies, Health Human Resources Research Center, School of Management and Medical Informatics, Shiraz University of Medical Sciences, Shiraz, Iran; 4. Assistant Professor, Research Center for Health Management in Mass Gathering, Red Crescent Society of the Islamic Republic of Iran, Tehran, Iran; 5. MPH of Health Policy, Health Policy Research Center, Institute of Health, Shiraz University of Medical Sciences, Fars, Iran

**Keywords:** COVID-19, cyberspace, disaster, health, social media

Dear Editor,

New Coronavirus (COVID-19) was first identified in Wuhan, Hubei Province, China in December 2019. This new COVID-19 is highly contagious with a high risk of infection, and due to its highly pandemic features, the World Health Organization (WHO; Geneva, Switzerland) declared a global health emergency.^1^ Social networks have a significant effect on management of diseases. Social networks are internet-based applications and include web-based and mobile technologies in which the user is able to create and exchange the contents that are used to transform communications into interactive conversations.^2^ An important advantage of social networks is the quick access to information. Social media could also be used to support the public health response to COVID-19. For example, during the quarantine period in China due to the COVID-19 outbreak, social networks found a proper opportunity to talk about the reasons for the quarantine, provide practical advice for people, and manage the rumors. Besides, various health recommendations, such as washing hands regularly, and other public health measures were communicated through the social networks.^3^ In Iran, the first positive COVID-19 case was reported by the Ministry of Health (Tehran, Iran) on February 18, 2020. From the first days of the COVID-19 outbreak in Iran, the social media actively managed to inform the public about the disease. Two social networks (ie, Telegram [Telegram Messenger LLP; London, United Kingdom] and Instagram [Facebook Inc.; Menlo Park, California USA]) had the most significant impact on the audiences. The social networks and cyberspace in Iran have had both positive and negative aspects during the counter with COVID-19, which have been discussed below.

The positive aspects included: (1) presentation of the realistic aspects and features of COVID-19; (2) outreaching of social media over traditional media such as television; (3) high speed of providing information; (4) continuation of high school and university education virtually; (5) performance of COVID-19 screening tests virtually; (6) public education; and (7) performance of virtual medical consultations.

The negative aspects included: (1) zooming in and out about the importance of COVID-19; (2) dissemination of unscientific news, rumors, and pseudoscience; (3) creation of terror among people; (4) creation of a negative psychological atmosphere in the society; (5) stimulation of negative emotions; (6) character assassination of the scientific and official figures; (7) creation of ethnic and religious divisions; (8) selling of counterfeit drugs through the cyberspace; and (9) spread of rumors about the infection of famous athletes and executive and political figures with COVID-19.

The following measures are suggested for the better management of the cyberspace in Iran: (1) increasing the public’s media literacy; (2) maintaining ethical frameworks by the media and the public; (3) monitoring the cyberspace continuously; (4) filtering and blocking the channels and groups spreading rumors; (5) encouraging people to use local apps; (6) transferring non-native application servers to other countries; (7) securing the cyberspace; (8) creating a national information network; (9) making the public inconsiderate to rumors; (10) developing crisis and disaster communication rules and regulations; (11) making the public resistant against disappointing news about COVID-19; (12) discovering and responding quickly to rumors and treating the rumormongers seriously; and (13) increasing trust in the media and governmental virtual networks.

## Conclusion

Since COVID-19 is a pandemic and its prevalence is increasing in Iran, managing the cyberspace has been regarded as a major challenge. The cyberspace looks like a double-edged sword with both positive and negative effects. Hence, national and local governments are required to monitor the cyberspace and determine the boundaries of social responsibility and professional-ethical framework of the media during crises and disasters to prevent the spread of any chaos and false information in the cyberspace. Additionally, social media and health planners and policymakers should collaborate to create realistic and scientific information in the cyberspace. Proper management of the cyberspace makes the public feel relaxed, reassures people to give a better response to COVID-19, and helps return the conditions to the pre-pandemic status as quickly as possible.
